# Books and Magazines

**Published:** 1901-05

**Authors:** 


The Technique of Surgical Gynecology.—By Prof. A. H. Goelet, Professor of Gynecology in the New York School of Clinical Medicine, Consulting Professor in Gynecological Electro-Therapeutics, International School of Correspondence, Scranton, Pa.; Fellow of the New York Academy of Medicine, and of the New York Obstetrical Society; Member of the American Medical Association, New Yory County Medical Association; Fellow of the Societe Francais d'Electro-Therapie, etc. 340 pages; 150 original drawings; bound in cloth; printed in white leaf. New York: International Journal of Surgery Co. 1901. Price, $2.00.

Leaving out of consideration the element of personal skill, the success of a gynecological operation depends not only upon an accurate acquaintance with the technic, but also upon a thorough knowledge of the many details connected with the preparation of a case for operation and with the after-management. Of all these important points this book affords complete and explicit information. Commencing with the preparation of the patient and of the field of operation the author describes minutely the preparation of the operator, assistants, nurses, of the operating room, instruments, dressings, etc. Then follow detailed and clear descriptions of each operation illustrated with a profusion of original drawings and photographs which still further elucidate the text. The concluding chapters of the book are devoted to the no less important subject of the after-care of the patient. In view of the fact that this work contains a large amount of valuable information not to be found in the text-books on the diseases of women, it will serve as a practical guide to every one desirous of acquiring a knowledge of the technique of surgical gynecology.

---

A SYSTEM OF PRACTICAL THERAPEUTICS.—By eminent American and foreign authorities. Edited by Hobart Amory Hare, M. D., Professor of Therapeutics, Jefferson Medical College; Physician to Jefferson College Hospital, etc., Philadelphia. New (2nd) edition, thoroughly revised. In three very handsome octavo volumes, containing 2593 pages, with 427 engravings, and 26 full-page colored plates. Per volume, cloth, $5.00, net; leather, $6.00, net; half morocco, $7.00, net. Lea Brothers & Co., Publishers, Philadelphia and New York. 1901.

The three volumes are now ready for delivery.

This system is designed to furnish a thoroughly practical work of reference in medical treatment, and also in the management of such surgical cases are are met with by every physician. The needs of the general practitioner have been kept constantly in view by the editor and his collaborators, and their endeavor has been to prepare articles so clear and definite, so comprehensive and detailed that the reader may be able to carry out successfully the methods which the widest experience has shown to produce the best results.

The work is above all practical. Each author tells with minute detail how he would treat the case under consideration if he himself were at the bedside. Illustrations have been freely used whenever they can make the text more clear, and prescriptions indicating the best methods for combining remedies for definite purposes will be found in abundance throughout the work. Remedial agents other than drugs, preevntive measures, etc., are carefully and completely covered, and in the third volume special attention is given to treatment in those general and special surgical affections which the family physician is likely to meet in his regular practice.

Although nominally a second edition this system is practically a new work, having been carefully revised in every line in order to reflect the knowledge of today. Many of the articles are entirely new.

SELF-EXAMINATION FOR MEDICAL STUDENTS.—Containing 3500 questions, with references to answers, also the questions of the examining boards of Pennsylvania, New York and Illinois. Third edition. Price, 10 cents. P. Blakiston Son & Co., Publishers, Philadelphia.

---

THE MEDICAL NEWS POCKET FORMULARY.—New (3rd) edition, containing 1700 prescriptions representing the latest and most approved methods of administering remedial agents. By E. Quin Thornton, M. D., Demonstrator of Therapeutics, Pharmacy and Materia Medica in the Jefferson Medical College, Philadelphia. New (3rd) edition, carefully revised to date of issue. In one wallet-shaped volume, strongly bound in leather, with pocket and pencil. Price, $1.50, net. Lea Brothers & Co., Philadelphia and New York. 1901.

